# La maladie de Weber-Christian: s'agit-il d'un état pré-leucémique?

**DOI:** 10.11604/pamj.2019.32.127.16106

**Published:** 2019-03-18

**Authors:** Salahiddine Saghir, Toufik Meskini, Said Ettair, Naima Erreimi, Nezha Mouane

**Affiliations:** 1 Département d'Hépato-gastroentérologie et de Nutrition-P III, Université Mohammed V, Rabat, Maroc

**Keywords:** Maladie de Weber-Christian, nodosités sous cutanées, panniculite idiopathique, leucémie aigue, Weber-Christian's disease, subcutaneous nodules, idiopathic panniculitis, acute leukemia

## Abstract

La maladie de Weber-Christian ou panniculite idiopathique est une affection rare, caractérisée par une inflammation du tissu adipeux sous-cutané; il s'agit d'une entité pathologique non spécifique qui reste toujours un sujet de débat et dont l'évolution est imprévisible. Nous rapportons dans cet article le cas d'un nourrisson de sexe féminin de 9 mois, admise dans un tableau de sepsis précédé d'une symptomatologie respiratoire et suivi de l'apparition de nodosités sous cutanées érythémateuses, de petite taille, dures, sensibles, asymétriques et situées au niveau des bras et des membres inférieurs, la biopsie cutanée a montré une hypodermite lobulaire avec infiltration des polynucléaires neutrophiles. La maladie de Weber-Christian a été retenue après avoir éliminé les autres diagnostics différentiels. Notre patiente a été traitée par la prednisone avec une bonne évolution initiale. Durant le suivi et au cours de la dégression, le diagnostic d'une leucémie aigue lymphoblastique type B a été posé. Cette évolution atypique à notre connaissance n'a jamais été décrite dans la littérature. Nous allons discuter à travers ce cas les différentes situations similaires où un autre diagnostic a été fait après avoir pris en charge les malades comme ayant une panniculite lobulaire idiopathique, en concluant de la nécessité d'une enquête étiologique exhaustive et d'une surveillance prolongée afin de rechercher une éventuelle pathologie associée.

## Abstract (en)

Weber christian's disease or idiopathic panniculitis is a rare condition characterized by an inflammation of the subcutaneous adipose tissue; it is a non-specific pathological condition that remains a central issue of debate and whose outcome is unpredictable. We here report the case of a 9-month-old female infant admitted in the hospital with sepsis preceded by respiratory symptoms and followed by the occurrence of small, hard, susceptible, asymmetrical erythematous subcutaneous nodes located in the arms and lower limbs. Cutaneous biopsy showed lobular hypodermitis with polymorphonuclear neutrophil infiltrations. The diagnosis of Weber Christian's disease was retained after eliminating other differential diagnoses. The patient was treated with prednisone with good outcome; during the follow-up period and during disease regression the diagnosis of B-cell acute lymphoblastic leukemia was made; this uncommon outcome has never been described in the literature. The aim of this study is to discuss any similar situation where another diagnosis was made in patients initially treated for idiopathic lobular panniculitis. This highlights the need for exhaustive etiological investigations and prolonged monitoring in order to search for a possible associated disorder.

## Introduction

La panniculite est une inflammation du tissu adipeux sous-cutané ou hypodermite qui peut être aigue ou chronique, plusieurs causes sous-jacentes sont décrites, en fonction du type de l'atteinte histologique, malgré la ressemblance de la présentation clinique des différentes formes [1]. Nous rapportons dans ce manuscrit une évolution atypique décrite pour la première fois dans la littérature d'un cas de panniculite lobulaire idiopathique.

## Patient et observation

Nous rapportons le cas d'un nourrisson de 9 mois, de sexe féminin, 3^ème^ d'une fratrie de 3 (fratrie bien portante), issue d'un mariage consanguin de 1^er^ degré, sous allaitement mixte depuis la naissance jusqu'à l'âge de 6 mois, puis la diversification alimentaire était instaurée. Le développement psychomoteur et staturo-pondérale était normal. Aucune notion de prise médicamenteuse, ni de toxine n'a été noté. Le début des symptômes remonte à 3 semaines avant l'admission par la survenue d'une rhinorrhée claire associée à une toux sèche dans un contexte de fièvre chiffré à 39°C, la patiente a été mise sous amoxicilline donnée (50mg/kg) en ambulatoire sans amélioration, l'évolution a été marquée par l'apparition d'une hypotonie généralisée et d'une détresse respiratoire motivant une admission dans un milieu hospitalier. L'examen clinique à l'admission retrouve une fièvre à 38,5°C, une polypnée à 54c/min, une tachycardie à 160 battements/min, la tension artérielle à 9/5cmHg, la saturation en oxygène à 88%, le temps de recoloration inférieur à 3 secondes, les conjonctives étaient décolorées avec une pâleur cutanéo-muqueuse, une bouffissure du visage et un œdème palpébral, l'examen cardiovasculaire retrouvait un souffle systolique au foyer mitrale; des signes de lutte respiratoires avec des râles ronflants bilatéraux à l'examen de l'appareil respiratoire et une hépatomégalie à l'examen abdominale.

Le bilan paraclinique à l'admission a mis en évidence une anémie à 2,5g/dl d'hémoglobine, le volume globulaire moyen était à 70,8um^3^ et la concentration corpusculaire moyenne en hémoglobine est de 28% avec un taux de réticulocytes à 80.000/ul, les leucocytes étaient à 5520/ul et le taux des plaquettes à 97.000/ul, le frottis sanguin n'a pas montré la présence de cellules malignes, la protéine C réactive était à 310, la vitesse de sédimentation à 98mm, le bilan hépatique, rénale, lipidique et le bilan d'hémolyse étaient sans particularités, le taux d'albumine était de 24g/l, la protéinurie était négative, le myélogramme au niveau des 2 crêtes, la radiographie pulmonaire standard étaient sans particularité et l'échographie abdominale a objectivé une hépatosplénomégalie.

**Figure 1:**
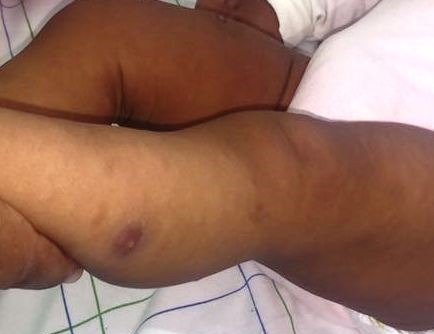
Nodosités sous cutanées dures et sensibles des membres inférieurs

**Figure 2:**
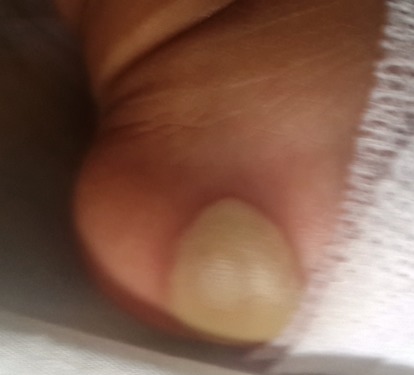
Évolution des nodosités vers la fluctuation

**Figure 3:**
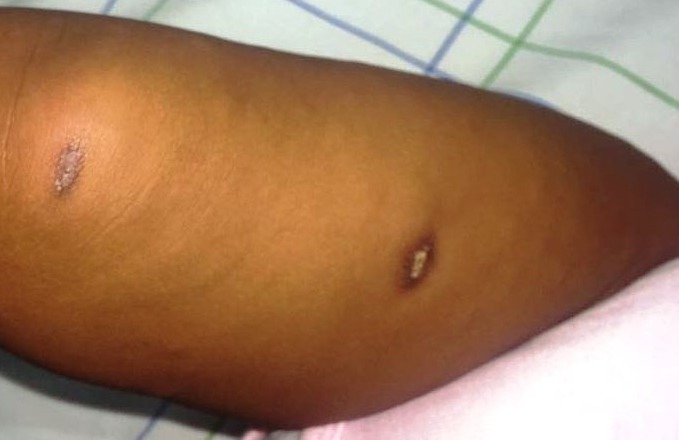
cicatrisation des lésions en laissant une hyperpigmentation et une dépression cutanée locale

La patiente a été mise sous bi-antibiothérapie à base de ceftriaxone 50mg/kg/j et de gentamycine 3mg/kg/j puis transfusée par un culot globulaire standard, suivie d'une disparition de la détresse respiratoire et d'une normalisation des valeurs de la saturation en oxygène. Après 48 heures d'évolution, l'apyrexie a été obtenue avec une disparition de l'œdème faciale, une persistance de l'encombrement bronchique et l'apparition de nodosités sous cutanées érythémateuses, de petite taille, dures, sensibles (Figure 1), situées au niveau des bras et des membres inférieurs, réparties d'une manière asymétrique, la face et le tronc ont été respectés, évoluant spontanément dans un intervalle de 4 jours vers la liquéfaction (Figure 2), puis la cicatrisation spontanée avec l'apparition d'une hyperpigmentation et d'une dépression cutanée locale (Figure 3), après émission d'un liquide huileux jaune citrin.

L'intradermoréaction à la tuberculine et les sérologies syphilitiques et virales étaient négatives, le bilan du déficit immunitaire était normal et la biopsie cutanée a montré une hypodermite lobulaire aiguë avec cystostéatonécrose sans vascularite, des infiltrats de polynucléaires neutrophiles et absence de cellules malignes et histiocytaires, l'examen bactériologique était négatif. L'électrophorèse des protéines a montré un syndrome inflammatoire aves une présence du pic a1, le bilan pancréatique était normal, les anticorps anti-DNA étaient négatifs et les anticorps anti-nucléaires étaient positifs (titre à 320). Le diagnostic de la maladie de Weber-Christian avec atteinte multisystémique a été retenu. Un bilan de contrôle à la fin de l'antibiothérapie et revenu négatif, y compris le bilan infectieux, l'échographie abdominale de contrôle était normale, alors qu'une deuxième poussée cutanée est réapparue, la patiente a été mise sous prednisone 2mg/kg/j. L'instauration du traitement a été marquée par la disparition rapide des manifestations cutanées. Le contrôle clinique et biologique à 2 semaines puis à 4 semaines était sans anomalies; la dégression progressive de la corticothérapie a été débutée.

A J15 de la première dégression, la patiente a été admise au service dans un tableau clinique similaire à celui de la première hospitalisation avec un syndrome anémique, une détresse respiratoire, un syndrome hémorragique cutanée (pétéchies) et une hépatosplénomégalie. Le bilan demandé a révélé un syndrome inflammatoire biologique, une anémie et une thrombopénie profonde, le frottis sanguin a objectivé la présence de 40% de blastes, un deuxième myélogramme qui a été completé par immunophénotypage a permis de faire le diagnostic de la leucémie aiguë lymphoblastique (LAL) type B et la patiente a été transferée au service d'oncologie pédiatrique pour chimiothérapie.

## Discussion

La maladie de Weber-Christian ou panniculite idiopathique est une affection rare, historiquement découverte par Pfeifer en 1892, puis mieux décrite sur le plan clinique et histologique par Weber et Christian en 1925 et en 1928 [2, 3]. Elle touche surtout l'adulte et est de prédominance féminine [4]. Plusieurs cas pédiatriques ont été cités, au Maroc seulement un cas est rapporté dans la littérature [5]. Elle se manifeste cliniquement par des poussées récurrentes de fièvre et nodules sous-cutanés douloureux, violacés sur un fond érythémateux, récidivants, de petit diamètre généralement de 1 à 2 cm, les lésions siègent surtout au niveau des membres inférieurs (cuisses, jambes, fesses), au niveau des membres supérieurs (la face postérieure des bras) et le tronc. Les lésions évoluent après un court délai vers le ramollissement, la fluctuation formant ainsi un kyste qui s'évacue en libérant un liquide jaune citrin stérile, laissant une cicatrice atrophique hyperpigmentée [4]. L'atteinte multisystémique comporte l'atteinte respiratoire (toux, dyspnée, douleur thoracique), cardiaque, rénale ( protéinurie, hématurie), hépatique et splénique conduisant à une hépatosplénomégalie, digestive (douleur et distension abdominale, diarrhée, hémorragie digestive, atteinte mésentérique), rarement neurologique (l´hémorragie cérébelleuse); ces formes sont associées à un taux de morbidité et de mortalité élevé [6].

Le bilan biologique retrouve essentiellement un syndrome inflammatoire (élévation de la vitesse de sédimentation, hypergammaglobulinémie, anémie inflammatoire), les formes systémiques s'accompagnent de manifestations biologiques en fonction de l'organe atteint. Le diagnostic de la maladie de Weber-Christian a été retenu chez notre patiente après élimination de toutes les étiologies des panniculites lobulaires avec une infiltration neutrophile (cause enzymatique, déficit en a1 antitrypsine, panniculite infectieuse). L'atteinte était multisystémique car le nourrisson présentait un encombrement respiratoire, une hepatosplenomégalie et une bicytopénie signalant une atteinte médullaire, la positivité des anticorps antinucléaires renforce l'hypothèse dysimmunitaire du mécanisme physiopathologique [7].

La panniculite neutrophile (PN) est une entité clinico-histologique qui regroupe plusieurs dermatoses caractérisées par des manifestations cliniques presque similaires, elles présentent des points communs et peuvent s'associer à des maladies de système et à des hémopathies. En effet la PN peut précéder et même suggérer l'éventualité d'un syndrome myélodysplasique (SMD) sous-jacent qui peut apparaitre même au cours du suivi clinique ou être un signe annonciateur d'une progression vers une leucémie aigüe myéloïde (LAM) [8]. Cette association entre PN et SMD est significativement élevée avec un pourcentage de 75% dans une série de 8 cas [9].

Le diagnostic différentiel, surtout en présence d'une symptomatologie respiratoire initiale dans notre cas, se fait essentiellement avec l'érythème noueux qui peut être aussi révélateur d'une hémopathie maligne sous-jacente surtout la LAM. Ceci est fréquemment rapportée dans la littérature [10, 11], et aussi avec le syndrome de Sweet dont la pathologie néoplasique associée chez l'enfant dans 14% des cas est la LAM [12]. L'étude anatomopathologique dans notre contexte a permis d'éliminer ces diagnostics en montrant une atteinte strictement lobulaire sans leucocytoclasie.

Un syndrome d'activation macrophagique précédant la survenue de la leucémie était également discutée mais on ne disposait que de 3 critères diagnostiques à la place de 5 afin de le retenir [13] et la régression des symptômes sous antibiothérapie et avant l'instauration des corticoïdes n'était pas en faveur.

La maladie de Weber-Christian (MWC) ou panniculite lobulaire fébrile récidivante idiopathique, est fréquemment citée dans la littérature chez l'adulte et rarement chez l'enfant, mais selon la plupart des auteurs il s'agit d'une entité non spécifique qui englobe probablement d'autres types plus spécifiques de panniculite. Cette approche dite idiopathique reste toujours un sujet de débat, selon l'étude de White JW Jr *et al*. [14], d'autres diagnostics plus précis ont été établis chez 30 patients qui étaient considérés initialement comme ayant une MWC. Concernant la population pédiatrique, dans la série de Fang Wu *et al.* [6], le diagnostic était admis sur des données cliniques et histologiques après élimination des autres étiologies mais avec un taux de mortalité élevée (45%). Malgré un traitement bien codifié, nous suggérons qu'une réévaluation des patients pourrait être bénéfique et aboutir à d'autres éventualités pathologiques.

Dans notre cas, le suivi de la patiente a permis de dépister une LAL qui était prise au début comme une deuxième poussée de la MWC. A notre connaissance, il s'agit du premier cas rapporté dans la littérature, le défi principal réside dans la découverte d'un lien de causalité entre cette dermatose et la leucémie, car les manifestations cutanées peuvent être un élément précurseur d'une maladie maligne sous-jacente. La corticothérapie est le traitement de choix des panniculites idiopathiques [15]. Il permet de contrôler la poussée et de faire dissoudre les nodules dermatologiques dans la plupart des cas, mais le début du traitement pourrait masquer une maladie sous-jacente pendant un certain temps, ce qui explique le délai de 6 semaines avant l'apparition des nouveaux symptômes (2 semaines après la dégression). La recherche d'un diagnostic différentiel et d'une pathologie associée doit être intensive avant le traitement. Elle était négative dans notre cas.

## Conclusion

Bien que rare, l'association d'une panniculite neutrophile de type érythème noueux ou syndrome de Sweet à une leucémie aigüe surtout myéloïde est décrite aussi bien chez l'adulte que chez l'enfant, alors qu'une telle situation n'a jamais été décrite dans le cadre de la maladie de Weber-Christian. Devant cette présentation clinicobiologique et histologique, une enquête étiologique exhaustive et une surveillance prolongée sont impératives afin de rechercher une éventuelle pathologie associée.

## Conflits d’intérêts

Les auteurs ne déclarent aucun conflit d'intérêts.
